# Tet2-mediated epigenetic drive for astrocyte differentiation from embryonic neural stem cells

**DOI:** 10.1038/s41420-020-0264-5

**Published:** 2020-04-29

**Authors:** Fei He, Hao Wu, Liqiang Zhou, Quan Lin, Yin Cheng, Yi E. Sun

**Affiliations:** 1 Shanghai Institute of Stem Cell Research and Clinical Translation, Shanghai East Hospital, Tongji University School of Medicine, Shanghai, 200092 China; 2 Department of Genetics, University of Pennsylvania, Philadelphia, PA 19104 USA; 3 Penn Epigenetics Institute, University of Pennsylvania, Philadelphia, PA 19104 USA; 4 Department of Psychiatry and Biobehavioral Sciences, Intellectual Development and Disabilities Research Center, David Geffen School of Medicine, University of California, Los Angeles, CA 90095 USA; 5 Collaborative Innovation Center for Brain Science, Tongji University, Shanghai, 200092 China

**Keywords:** Cellular neuroscience, Neural stem cells

## Abstract

DNA methylation and demethylation at CpG di-nucleotide sites plays important roles in cell fate specification of neural stem cells (NSCs). We have previously reported that DNA methyltransferases, Dnmt1and Dnmt3a, serve to suppress precocious astrocyte differentiation from NSCs via methylation of astroglial lineage genes. However, whether active DNA demethylase also participates in astrogliogenesis remains undetermined. In this study, we discovered that a Ten-eleven translocation (Tet) protein, Tet2, which was critically involved in active DNA demethylation through oxidation of 5-Methylcytosine (5mC), drove astrocyte differentiation from NSCs by demethylation of astroglial lineage genes including Gfap. Moreover, we found that an NSC-specific bHLH transcription factor Olig2 was an upstream inhibitor for Tet2 expression through direct association with the Tet2 promoter, and indirectly inhibited astrocyte differentiation. Our research not only revealed a brand-new function of Tet2 to promote NSC differentiation into astrocytes, but also a novel mechanism for Olig2 to inhibit astrocyte formation.

## Introduction

Neural stem cells (NSCs) are self-renewing, multipotent stem cells that possess both the ability to proliferate and self-renew and to differentiate into three major cell lineages in the central nervous system (CNS), namely neurons, astrocytes, and oligodendrocytes^1^. Lineage differentiation into these three cell types is tightly regulated in a spatial and temporal-specific manner^2,3^. Both in vivo and in vitro, NSCs first differentiate into neurons then glial cells^3,4^. In mouse cortex, neurons are mainly produced between embryonic day E10.5 to E16, astrocytes are mainly produced between E16 to postnatal stages, peaking around P3, and oligodendrocytes are mainly generated postnatally^5^. Mouse cortical NSCs dissected from different embryonic stages also show different differentiation potentials in vitro. NSCs dissected from early embryonic stages (E11-12) mainly give rise to neurons, even in the presence of glial induction factors (e.g., bone morphogenetic protein (Bmp) or leukemia inhibitory factor (LIF)). During in vitro culturing, NSCs gradually acquire competence for astrogliogenesis and dampen their neurogenic potential, indicating the existence of an intrinsic switch mechanism from neurogenic to astrogliogenic^1,5^.

While extrinsic environmental signals and specific transcription factor and co-factor networks are important in regulating cell fate^2,3,6^, epigenetic modifications, such as histone modifications, DNA methylation, chromatin remodeling, and non-coding RNAs are also crucial in mediating the proper regulation of developmental stage-specific gene expression^7–10^. DNA methylation as one of the major epigenetic mechanisms has been previously postulated to regulate cell fate specification of NSCs^7^ and control the sequential generation of neurons and glia^6^. Our previous work demonstrated that a de novo DNA methyltransferase, Dnmt3a, also plays a key role in maintaining neurogenesis and preventing premature astrogliogenesis in early NSCs^11^. In addition, we showed that enhanced Jak-Stat signaling as well as early astrogliogenesis in NSCs lacking the maintenance DNA methyltransferase Dnmt1^1^. Studies by others have shown that the methylation at the promoter of Gfap, a canonical astrocyte marker, is anti-correlated with the expression of Gfap as well as astrogliogenesis, and DNA methylation inhibits activation of gliogenesis through hypomethylation at promoters of astroglial lineage genes (e.g., Gfap, S100b)^8,12,13^. Moreover, Nakashima et al. analyzed DNA methylation changes in mouse NPCs between mid (E11.5) and late (E14.5) stages of cortical development and found that many astrocytic genes, including Gfap become demethylated in late-stage NPCs, which then enables cells to become competent for astrogliogenesis^3^.

DNA methylation was once considered to be an irreversible DNA modification, which can only be removed passively through cell division. Recent studies have shown that DNA methylation is highly dynamic and the regulation of DNA methylation plays important role during development and maturation of the CNS^11,14–17^. In addition to the maintenance DNA methylatransferase, Dnmt1, the developing CNS also expresses a de novo DNA methylatransferase, Dnmt3a, which is involved in adding methyl-Cytosine into unmethylated specific CpG sites in the genome. From the DNA demethylation perspective, there are three members in the Tet family, Tet1, Tet2, and Tet3. The Tet family members, which are involved in the process of active DNA demethylation, and have been implicated in embryonic development and neural lineage differentiation^17,18^. Here, we report a novel mechanism by which Tet2 drives embryonic NSC towards astrocytic lineage differentiation through epigenetic regulation, and that Olig2 serves as an upstream inhibitor for Tet2 expression to also indirectly suppress astroglial lineage differentiation.

## Results

### Involvement of Tet2 in astrocyte differentiation from embryonic NSCs

**Fig. 1:**
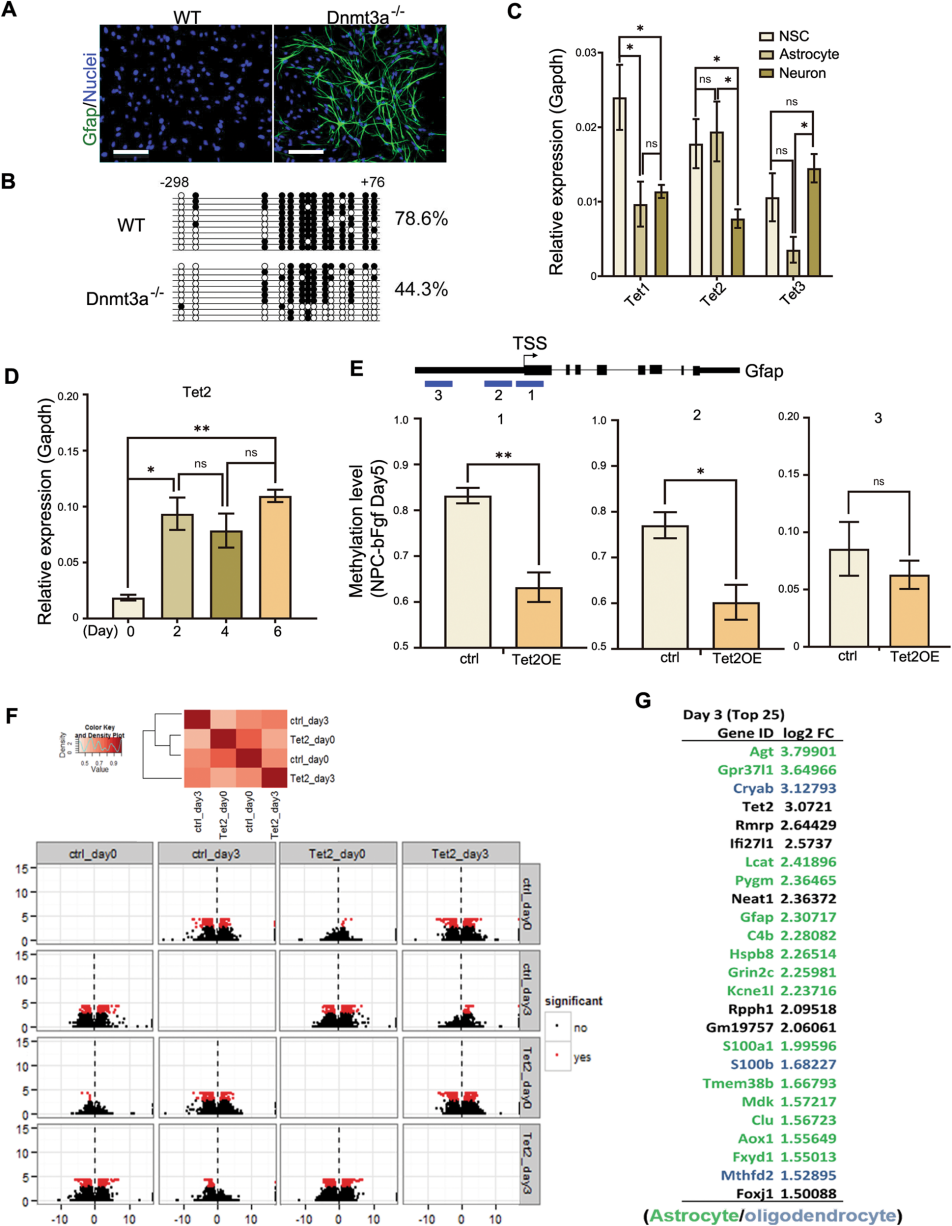
Tet2 positively regulates the expression of astroglial lineage genes. **a** Spontaneous differentiation of astrocytes from Dnmt3a-/- embryonic NSCs in the presence of bFGF. Scale bars, 100 μm. **b** DNA demethylation of the GFAP proximal promoter in Dnmt3a-/- NSCs. **c** Tet 1, 2, 3 mRNA expression in undifferentiated NSCs, fully differentiated astrocytes, and immature neurons isolated from E15 mouse cortices (Tet1, NSC vs astrocyte, *p* = 0.030; NSC vs neuron, *p* = 0.032; Tet2, NSC vs neuron, *p* = 0.044; astrocyte vs neuron, *p* = 0.039; Tet3, astrocyte vs. neuron, *p* = 0.037; *n* = 12, 3 independent biological repeats. One-way ANOVA, LSD test, *p < 0.05, ns, nonsignificant, Mean ± SD). **d** Tet2 mRNA levels during NSC spontaneous differentiation after bFGF withdrawal at Day 0, 2, 4, and 6. (Day2 vs Day0, *p* = 0.028; Day6 vs Day0, *p* = 0.0044; *n* = 12, 3 independent biological repeats. One-way ANOVA, Tukey test, *p < 0.05, **p < 0.01, ns, nonsignificant, Mean ± SD). **e** Exogenous Tet2 catalytic domain overexpression (Tet2OE) induced demethylation of Gfap promoter. Methylation levels at two proximal promoter segments near the TSS (1, 2) and distal promoter segment (3) in undifferentiated NSCs with control and Tet2OE conditions (ctrl vs. Tet2OE (1) *p* = 0.0044; (2) *p* = 0.021; *n* = 15, from three independent biological experiments (Two-tailed Student’s *t*-test, *p < 0.05, **p < 0.01, ns, nonsignificant, Mean ± SD). **f** Top: A Pairwise comparisons with Pearson correlation coefficients of transcriptomes from control and Tet2OE before and after differentiation at day 3. Red indicates a high similarity (cor = 1) and white indicates a lower similarity (cor = 0) between the two samples. Bottom: CummeRbund volcano plot reveals genes that are significantly differentially expressed between each pair. **g** Top 25 upregulated genes by Tet2OE in NSCs at day 3 following differentiation. Green indicates astrocyte lineage genes; blue indicates oligodendrocyte lineage genes and red indicates neuronal genes.

NSCs isolated from embryonic day 11 (E11) mouse forebrain and cultured in the presence of a mitogen, basic fibroblast growth factor (bFGF), normally remain undifferentiated (Fig. 1a). However, when a *de novo* DNA methyltransferase, Dnmt3a was knocked out from NSCs, spontaneous astrocyte differentiation occurred even in the presence of bFGF (Fig. 1a). Concurrently, DNA demethylation was observed within the promoter of a classic astroglial lineage gene, Gfap (Fig. 1b). This is somewhat unexpected, because usually maintenance of DNA methylation is conducted by Dnmt1, and in Dnmt3a KO NSCs, Dnmt1 remains intact. The fact that Dnmt3a is required to keep the Gfap proximal promoter in a highly methylated state in undifferentiated E11 (early) NSCs suggested that this locus is under the dynamic active methylation and demethylation controls, and its methylation could not be simply maintained by Dnmt1. To explore the potential active demethylation control of the astroglial lineage gene Gfap, we examined expression levels of Tet1, 2, and 3 in undifferentiated NSCs, fully differentiated astrocytes, and neurons isolated from E14/15 embryonic mouse cortex (Fig. 1c). We found only Tet2 remained high level expression in astrocytes as compared to NSCs, suggesting Tet2 function is compatible with astrocytic lineage. We further studied Tet2 expression in primary cultures of E11 NSC at different time points following spontaneous differentiation after bFGF withdrawal (Fig. 1d). It appears that the expression pattern of Tet2 is consistent with its potential role in regulating astroglial lineage differentiation.

To determine whether Tet2 may be involved in regulating the classic glial lineage gene Gfap promoter methylation, we performed Methylated DNA Immunoprecipitation (MeDIP) and quantitative PCR of three different segments of the Gfap promoter in E11 NSCs with or without overexpression of Tet2 catalytic domain (Tet2OE). We found that DNA methylation levels at the proximal promoter and transcription start site (TSS) (−206 bp to +426 bp) (both segments 1 and 2) decreased significantly in Tet2OE condition (Fig. 1e), while the distal segment (−1550 bp to −1350 bp), segment 3, only showed a trend of reduction, but did not reach statistical significance (Fig. 1e). Together these data suggest that Tet2 mainly targets the proximal promoter region of Gfap.

To further investigate the biological impact of Tet2OE in E11 NSCs, we performed unbiased genome-wide transcriptomic analyses. Primary E11 NSCs were infected with control or Tet2 catalytic domain overexpressing (Tet2OE) viruses and cultured with bFGF till 7 DIV (days in vitro). The cells were then subjected to spontaneous differentiation upon bFGF withdrawal. Samples at Day 0 and Day 3 following bFGF withdrawal were collected for RNA-sequencing. A general clustering of all samples based on Pearson correlation coefficients of the transcriptomes showed that gene expression changes after spontaneous differentiation as well as changes between control and Tet2OE (Fig. 1f). Comparing day 3 Tet2OE and day 3 control samples, the expression of 39 genes were significantly altered (Fig. 1f). Interestingly, all of them were upregulated by Tet2OE and majority of the top25 upregulated genes were astrocytic lineage specific genes based on work published by Ben Barres’s group^19^. There were some oligodendrocyte specific genes and very few neuronal genes (Fig. 1g).

**Fig. 2:**
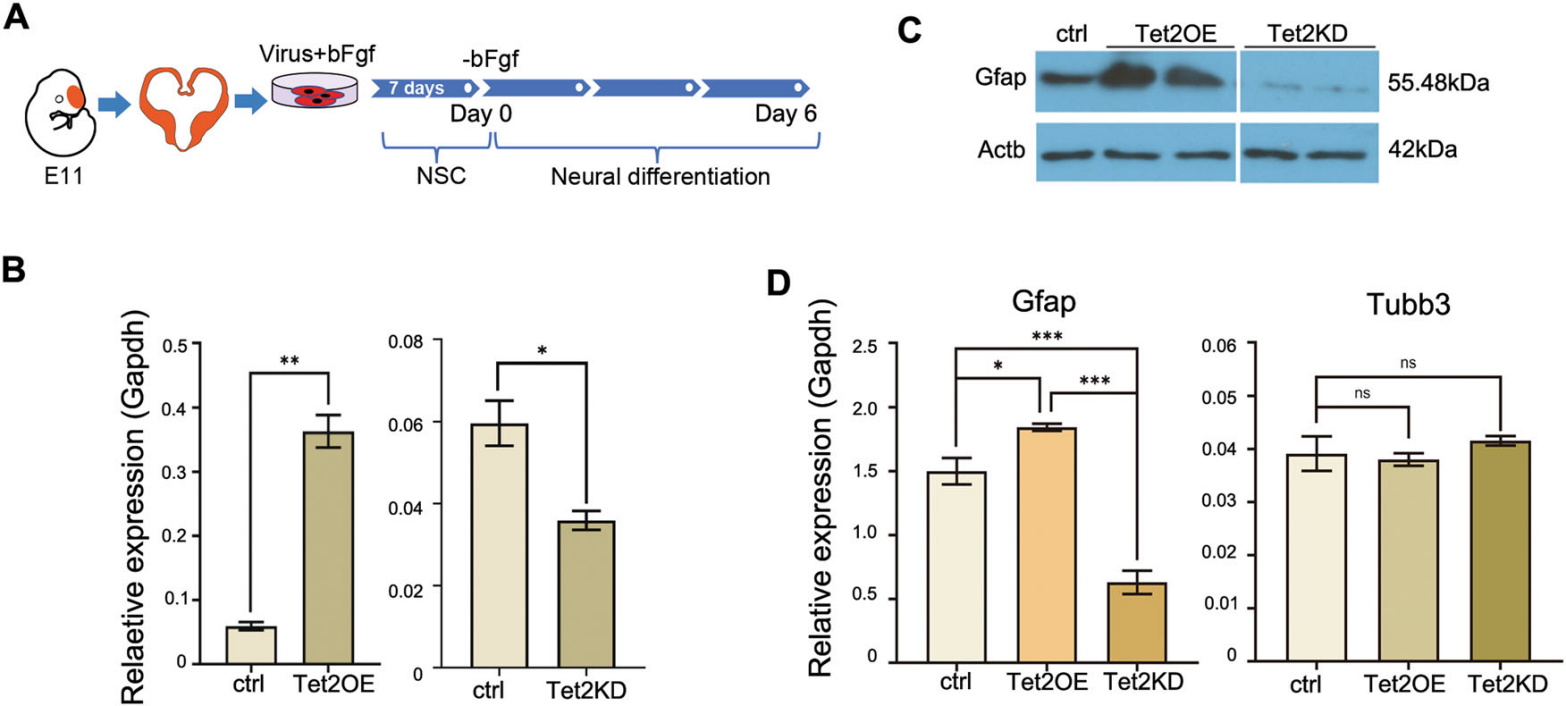
Tet2 promotes astrocyte differentiation. **a** a schema depicting the experimental design. **b** qRT-PCR of Tet2 demonstrating the extents of overexpression and knockdown of Tet2 (ctrl vs. Tet2OE, *p* = 0.0073; ctrl vs. Tet2KD, *p* = 0.0161; *n* = 12 from three independent biological experiments. Two-tailed Student’s *t*-test, *p < 0.05, **p < 0.01, Mean ± SD). **c** Representative Western blot analysis of Gfap protein levels in Tet2KD and Tet2OE NSCs, 7 days after differentiation. The beta-Actin was used as the internal control. **d** Quantitative PCR analysis of lineage markers Gfap, Tubb3, mRNA levels in control (scrambled), Tet2 overexpressed-NSCs (Tet2OE) and Tet2 knocked-down NSCs (Tet2KD), 7 days after differentiation. (Gfap, ctrl vs. Tet2OE, *p* = 0.041; ctrl vs. Tet2KD, *p* = 0.0007; Tet2KD vs. Tet2OE, *p* = 0.0001; *n* = 15, from three independent biological repeats. One-way ANOVA, Tukey test, *p < 0.05, ***p < 0.001, ns, nonsignificant, Mean ± SD).

Unbiased transcriptomic analyses suggested that Tet2OE promoted astrogliogenesis, to confirm this, we compared differentiation potential of E11 NSCs infected with control, Tet2OE, and Tet2 shRNA knock-down (Tet2KD) viruses. The extent of overexpression or knockdown was determined by quantitative RT-PCR (Fig. 2a–b). Exogenous Tet2OE promoted astrocyte lineage differentiation from NSCs (Fig. 2b–c) and conversely, Tet2KD inhibited astrocyte differentiation (Fig. 2b–c). Western blot analyses further confirmed immunocytochemical results (Fig. 2c). Quantitative RT-PCR further indicated that Tet2OE increased and Tet2KD decreased Gfap expression while having no effect on the expression of a neuronal marker, Tubb3 (Fig. 2d), indicating that in cultured E11 NSCs, Tet2 has little impact on neuronal lineage differentiation.

#### Olig2 is an upstream inhibitor for Tet2

While dynamic changes in DNA methylation is crucial for epigenetic regulation of cell fate specification of NSCs, proper regulation of the intracellular signaling and transcription factor pathways are essential for the transition from neurogenesis to astrogliogenesis^7,20^. It has been previously reported that Olig2 is crucial for the generation of motor neurons and oligodendrocytes in the CNS, depending on whether or not neurogenin 2 (Ngn2) is present^21^. Moreover, the phosphorylation status of Olig2 is also critical in regulating the proliferation of neural progenitors and the cell fate^22^. Besides its function in the motor neuron and oligodendrocyte lineage differentiation, Olig2 also acts to repress the astrocyte lineage in embryonic mouse cortex. Forced expression of Olig2 in NSCs lead to increased oligodendrocyte and decreased astrocyte differentiation both in vitro and in vivo^23,24^. However, how Olig2 inhibits astrocyte differentiation is not completely understood.

**Fig. 3:**
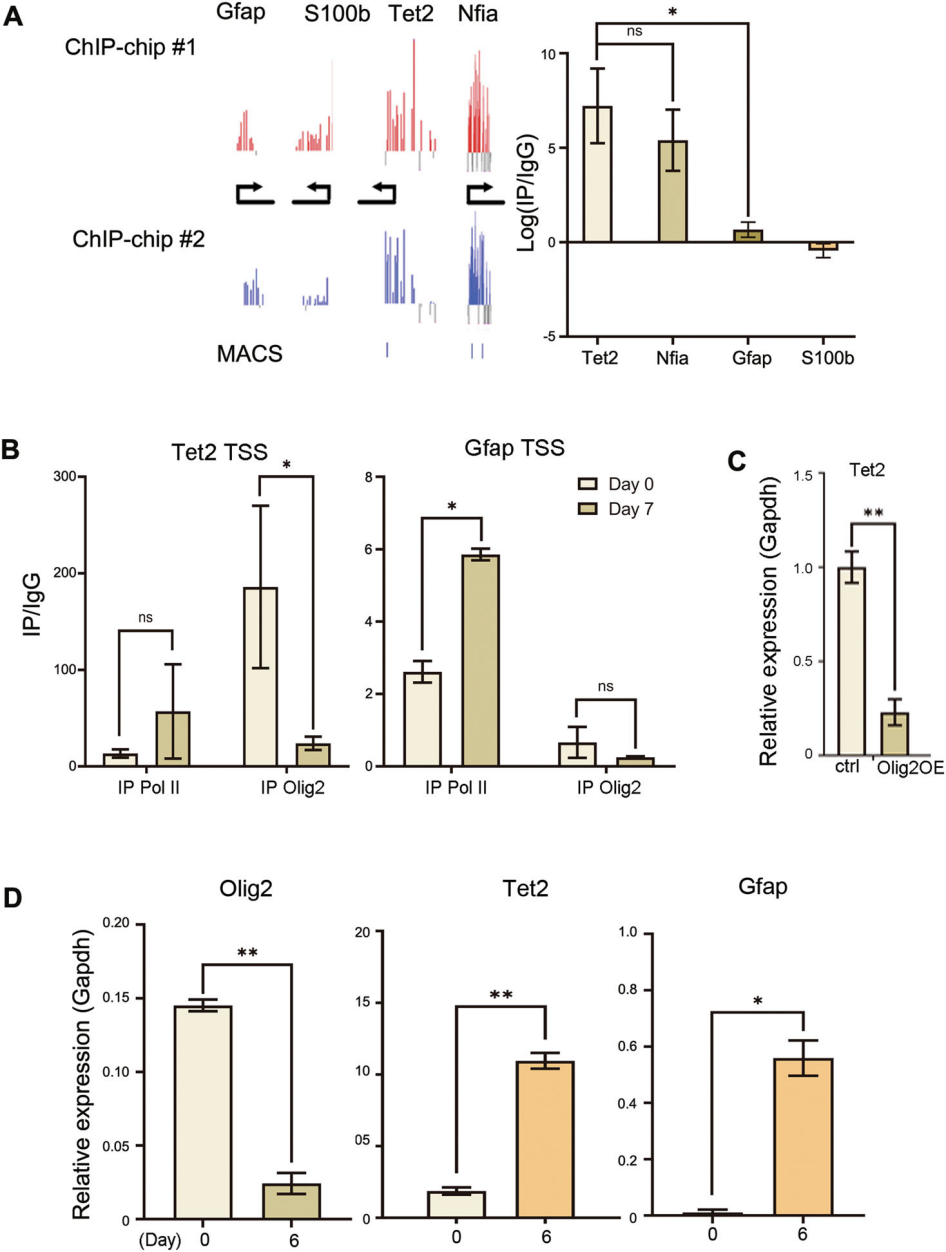
Olig2 binds to Tet2 promoter and represses the expression of Tet2. **a** ChIP-chip and ChIP-qPCR validation of Olig2 targets (Tet2, Nfia) and non-Olig2 targets (Gfap, S100b) in E11 cortical NSCs (Tet2 vs. Gfap *p* = 0.012, *n* = 15 from three independent biological repeats. Two-tailed Student’s *t*-test, *p < 0.05, ns nonsignificant, Mean ± SD). ChIP-chip replicates are shown in red and blue bars, the height of the bar plot indicates the enrichment of Olig2 at the corresponding locus, black arrow indicates the direction of transcription, blue bars at the bottom of the graphs indicates the true binding peaks identified by MACS. **b** Enrichment of RNA polymerase II, Olig2 at Tet2 and Gfap TSS during NSC spontaneous differentiation (IP Olig2, Tet2TSS, Day0 vs. Day7 *p* = 0.032. IP PolII, Gfap TSS, Day0 vs. Day7 *p* = 0.011, *n* = 6 from two independent biological repeats. Nonparametric *t*-test, *p < 0.05, ns nonsignificant, Mean ± SD). **c** Overexpression of Olig2 suppressed Tet2 gene expression (*p* = 0.0076, *n* = 15 from three independent biological repeats. Two-tailed Student’s *t*-test, **p < 0.01, Mean ± SD). **d** The expression of Olig2 anti-correlates with the expression of Tet2 and Gfap during astrogliogenesis (Olig2, *p* = 0.0024; Tet2: *p* = 0.0095; Gfap, *p* = 0.013. *n* = 12 from three independent biological repeats. Two-tailed Student’s *t*-test, *p < 0.05, **p < 0.01, Mean ± SD).

Olig2 is a basic helix-loop-helix (bHLH) transcription factor, with the bHLH domain binding to to E-box consensus sequence (CANNTG)^25,26^, and often functioning as a transcriptional repressor. We performed chromatin immunoprecipitation (ChIP) of Olig2 and preliminary genomic DNA tiling array (ChIP-chip) to identify potential binding targets for Olig2, which was further confirmed by ChIP-qPCR (Fig. 3a). ChIP-qPCR indicated that Olig2 directly associated with promoters of Tet2 and an astrocyte fate specification factor Nfia, but not promoters of Gfap or S100b, which are also astroglial lineage genes. Additional ChIP-qPCR assays indicated that upon NSC spontaneous differentiation, Olig2 reduced association with the Tet2 promoter (Fig. 3b), while a trend of increase in DNA polymerase II (Pol II) association with Tet2 promoter was seen, which is consistent with increased Tet2 expression (Figs. 3b, d and 1d). At the Gfap promoter, however, although Pol II association increased upon differentiation, which is consistent with increased Gfap expression, Olig2 does not appear to directly interact with the Gfap proximal promoter (Fig. 3b, d). To determine whether or not Olig2 association with the Tet2 promoter is inhibitory for transcription, we performed RT-qPCR analyses and found that Olig2 overexpression (OE) greatly inhibited Tet2 expression, consistent with a role of a transcription repressor (Fig. 3c). Lastly quantitative RT-PCR analysis showed that upon NSC spontaneous differentiation, the expression of Olig2 is anti-correlated to that of Tet2 and Gfap (Fig. 3d). Therefore, we hypothesize that Olig2 inhibits Tet2 expression to indirectly prevent precocious astrogliogenesis.

#### Olig2 inhibits astrogliogenesis and neurogenesis but not oligodendroglial lineage differentiation from E11 NSCs

**Fig. 4:**
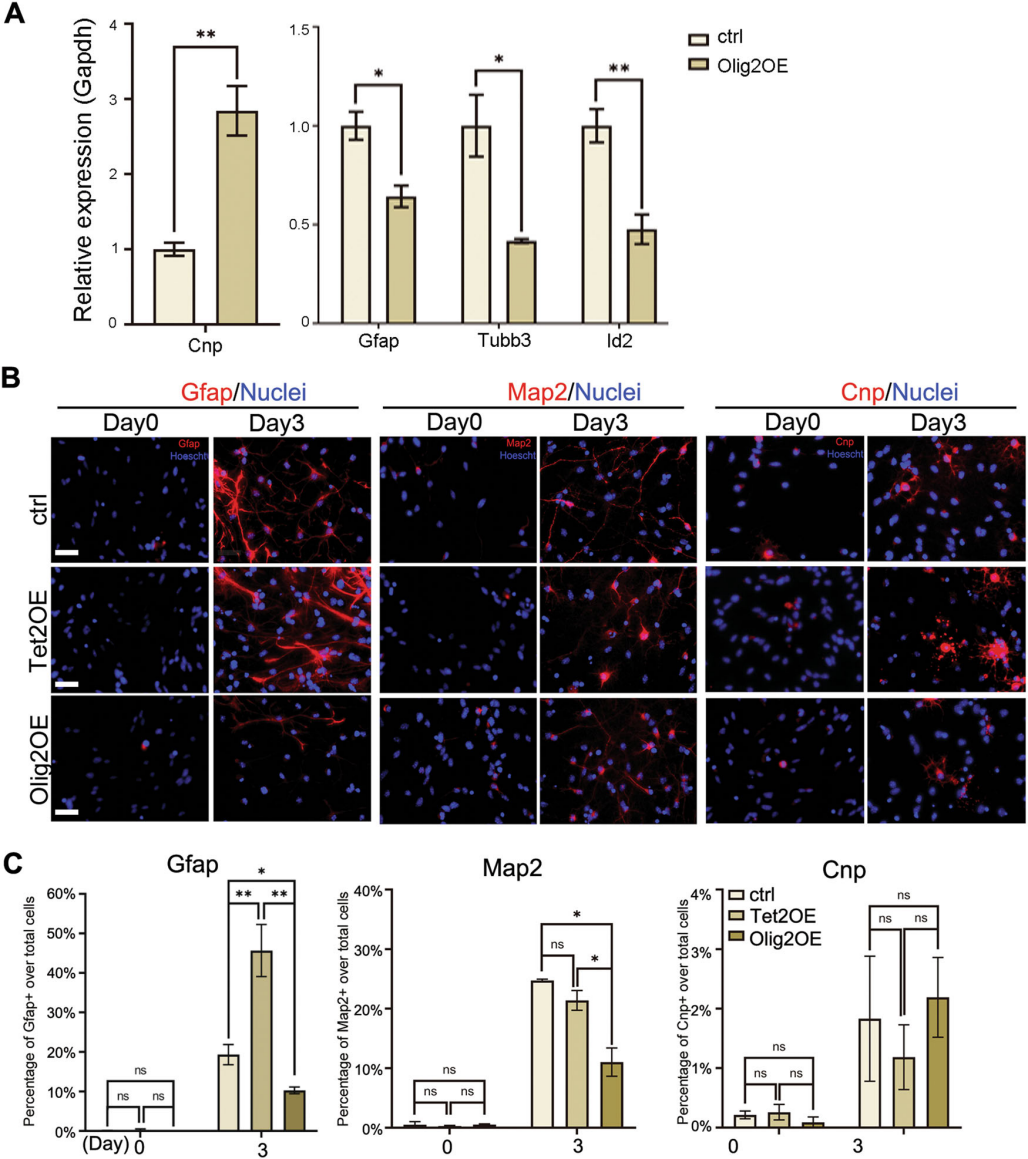
Olig2 and Tet2 regulated astrogliogenesis in an opposite manner. **a** Overexpression of Olig2 regulated neural lineage genes expression (Cnp, *p* = 0.0018; Gfap, *p* = 0.013; Tubb3, *p* = 0.031; Id2, *p* = 0.0035; *n* = 15 from three independent biological repeats. Two-tailed Student’s *t*-test, *p < 0.05, **p < 0.01, Mean ± SD). **b**, **c** Immunohistochemistry and quantification of Gfap+ astrocytes, Map2+ neurons, Cnp+ oligodendrocytes in ctrl and Tet2 or Olig2 overexpressed E11 NSCs (Gfap,ctrl vs. Tet2OE, *p* = 0.0091; ctrl vs Olig2OE, *p* = 0.0153; Tet2OE vs. Olig2OE, *p* = 0.0011; Map2, ctrl vs. Olig2OE, *p* = 0.0209; Tet2OE vs. Olig2OE, *p* = 0.0446, *n* = 18, three independent biological repeats. One-way ANOVA, Tukey test, *p < 0.05, ns nonsignificant, Mean ± SD). Scale bars, 50 μm.

To further investigate the biological function of Olig2 in regulating E11 NSC differentiation, lentiviral vectors carrying Olig2 overexpression cassette were introduced into primary E11 NSC cultures. Upon spontaneous differentiation, Olig2OE reduced Gfap and Id2 expression, both of which are involved in astrocyte differentiation. Olig2OE also inhibited expression of a neuronal marker Tubb3, but increased mRNA expression of an oligodendrocyte marker gene, 2′,3′-Cyclic-nucleotide 3′-phosphodiesterase (Cnp) (Fig. 4a). Immunocytochemical analyses indicated that Tet2OE promoted and Olig2OE suppressed astrocyte differentiation (Fig. 4b, c). Tet2OE did not affect, but Olig2OE inhibited neuronal differentiation from NSCs (Fig. 4b, c). Interestingly, Olig2OE elevated Cnp mRNA levels without increasing numbers of Cnp positive cells (Fig. 4b, c), suggesting the presence of post-transcriptional regulations of oligodendrocyte lineage genes, which has been reported before^27^.

#### Tet2 functions downstream of Olig2 in regulating astrogliogenesis

**Fig. 5:**
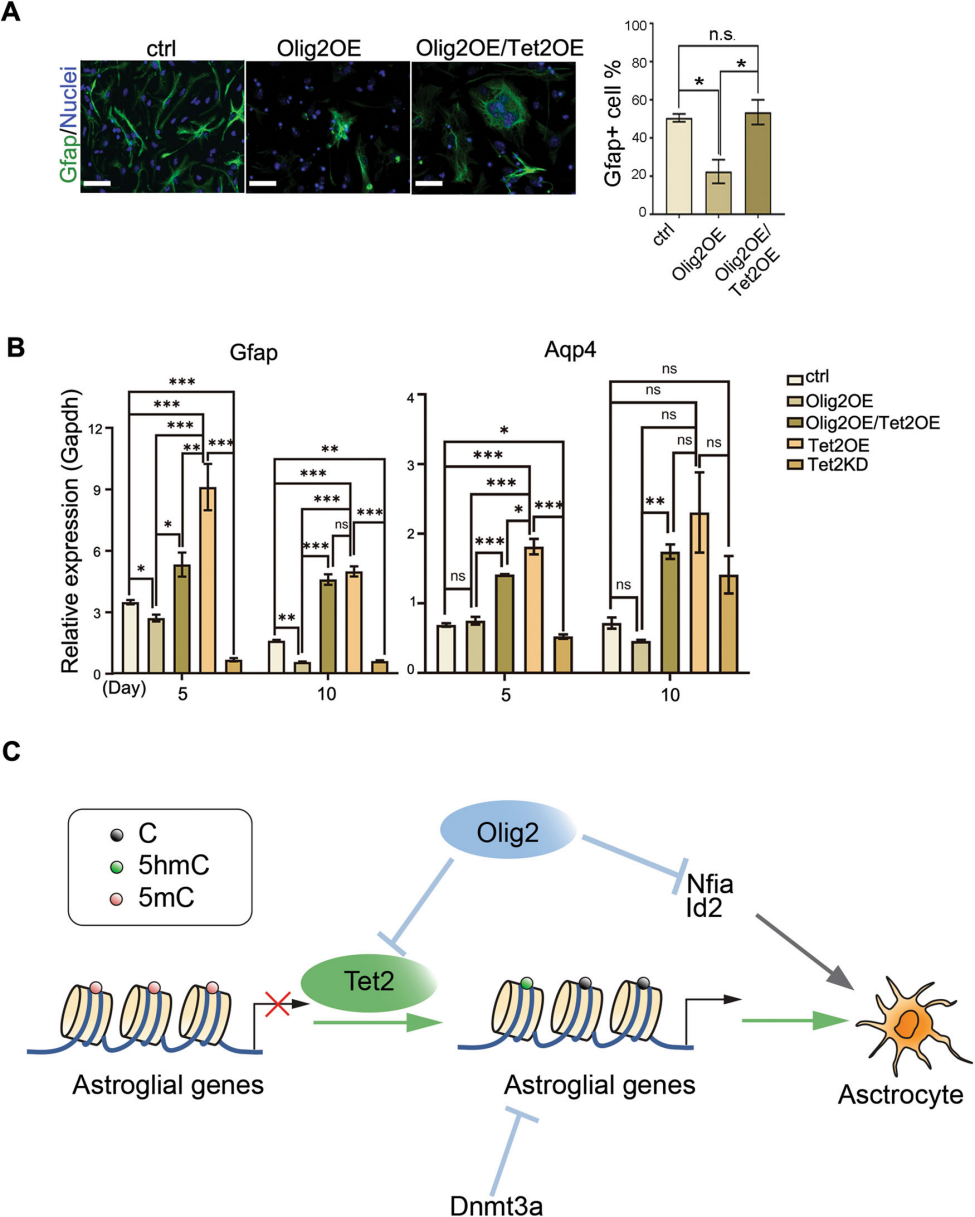
Tet2OE reverses inhibition of astrogliogenesis by Olig2OE. **a** E11 NSCs with Olig2OE and compound expression of Olig2 and Tet2, 6 days after differentiation (Gfap, ctrl vs. Olig2OE, *p* = 0.022; Olig2OE vs. Olig2OE/Tet2OE, *p* = 0.014, *n* = 15 from three independent biological repeats. One-way ANOVA, Tukey test, *P < 0.05, Mean ± SD). Scale bars, 50 μm. **b** Quantitative PCR analysis of astrocyte markers Gfap and Aqp4 mRNA levels in ctrl NSCs and NSCs with Tet2OE, Tet2KD, Olig2OE, and compound overexpression of Olig2 and Tet2, 5 and 10 days post differentiation. (Gfap, Day5, ctrl vs. Tet2OE, *p* = 0.00046; ctrl vs. Tet2KD, *p* = 0.00055; ctrl vs. Olig2OE, *p* = 0.031; Tet2OE vs. Tet2KD, *p* = 0.00032; Olig2OE vs. Olig2OE/Tet2OE, *p* = 0.026; Olig2OE/Tet2OE vs. Tet2OE, *p* = 0.0066; Olig2OE vs. Tet2OE, *p* = 0.0006. Day10, ctrl vs. Tet2OE, *p* = 0.00016; ctrl vs. Tet2KD, *p* = 0.0088; ctrl vs. Olig2OE, *p* = 0.0072; Tet2OE vs. Tet2KD, *p* = 0.00011; Olig2OE vs. Olig2OE/Tet2OE, *p* = 0.00006; Olig2OE vs. Tet2OE, *p* = 0.00005, *n* = 15 from 3 independent biological repeats. Two-way ANOVA, Tukey test, *p < 0.05, **p < 0.01, ***p < 0.001, ns, nonsignificant, Mean ± SD; Aqp4, Day5: ctrl vs. Tet2OE, *p* = 0.000065; ctrl vs. Tet2KD, *p* = 0.014; Tet2OE vs. Tet2KD, *p* = 0.000031; Olig2OE vs. Olig2OE/Tet2OE, *p* = 0.000294; Olig2OE/Tet2OE vs. Tet2OE, *p* = 0.029; Olig2OE vs. Tet2OE, *p* = 0.000632. Day10, Olig2OE vs. Olig2OE/Tet2OE, *p* = 0.0066, *n* = 15 from three independent biological repeats. Two-way ANOVA, Tukey test, *p < 0.05, **p < 0.01, ***p < 0.001, ns, nonsignificant, Mean ± SD). **c** Summary of Tet2 and Olig2 regulation of astrocytic differentiation. DNA demethylation by Tet2 is critical to initiate and establish the transcriptional program that promotes astrocyte differentiation. Olig2 not only represses the expression of astrocytic regulatory factors to block premature astroglial differentiation, it also directly represses Tet2 expression to indirectly maintain DNA hypermethylation astroglial genes.

To determine whether Olig2 inhibits astrocyte differentiation via inhibition of Tet2 expression, we performed compound overexpression experiments. Overexpression of Olig2 in cultured E11 NSCs reduced numbers of Gfap^+^ astrocytes, While Tet2OE together with Olig2OE significantly rescued the astrocyte differentiation phenotype (Fig. 5a). Moreover, quantitative PCR analyses of astrocyte markers Gfap as well as aquaporin 4 (Aqp4) (a blood-brain-barrier-associated astrocyte marker) mRNA levels further confirmed that Olig2 negatively regulates and Tet2 positive regulates astrogliogenesis from E11 NSCs (Fig. 5b). Moreover, epistatic analysis of Tet2 and Olig2 as well as observations described above, indicated that Tet2 function downstream of Olig2, and decreased expression of Olig2 could lead to upregulation of Tet2 and subsequent DNA demethylation of glial lineage genes to elicit astrogliogenesis (Fig. 5c).

## Discussion

Since the discovery of demethylation catalyzed by Tet family proteins, there have been extensive studies on their roles in gene expression regulation, embryonic development, and stem cell differentiation^17,28–32^. QPCR and western blot analysis showed that Tet2 was expressed at low levels in E11 NSCs, and its expression rapidly, yet transiently increased upon NSC differentiation in vitro. Overexpression of Tet2 promoted NSC differentiation towards astrocytic lineage, and had little effect on the differentiation towards neuronal or oligodendrocyte lineages. Depletion of Tet2 using shRNA knockdown significantly impaired NSC differentiation towards astrocyte lineage. These gain- and loss- of function studies strongly placed Tet2 as a key regulator of astroglial lineage differentiation.

It is worth mentioning that both maintenance and de novo DNA methyltransferase, Dnmt1 and Dnmt3a are also involved in suppressing precocious astroglial lineage differentiation by maintaining or adding methyl group to critical sites of CpG dinucleotide^1,11^. Therefore, Dnmts and Tet proteins provide “Yin” and “Yang” regulations of astroglial lineage gene methylation and astrocyte differentiation. This is somewhat of a surprise, because theoretically, through only regulation of Dnmt1, passive DNA demethylation of glial lineage genes could occur, which should be sufficient to trigger the developmental switch from neurogenesis to astrogliogenesis. Why Tet2 and Dnmt3a were brought to complicate the regulatory mechanism of astrocyte differentiation? Why biology does not follow the most parsimonious rule? The answer, we believe, lies in the importance of DNA methylation maintenance. Dnmt1 is the only maintenance DNA methyltransferase in the genome. Eliminating Dnmt1 expression could be detrimental. In fact, Dnmt1 conventional knockout is early embryonically lethal. Moreover, neural epithelial specific knockout of Dnmt1 is also perinatal lethal. Therefore, dynamic DNA methylation regulation must be more intricately conducted by both de novo DNA methyltransferase and Tet proteins. This is also true during early embryogenesis after fertilization regarding the demethylation and re-methylation of the paternal and maternal genome, where both *de novo* DNA methyltransferses and Tet proteins are both involved^1,8,13,17,33,34^. We have previously reported that DNA methylation at distal promoter region promotes neuronal lineage gene differentiation, whereas neuronal genes are under the negative epigenetic regulation by the PRC2 complex-mediated histone methylations. It seems that DNA methylation on astroglial lineage genes are mainly inhibitory, therefore as compared to neuronal and oligodendroglial lineages, DNA demethylation-related event is more important for initiation of astroglial lineage differentiation.

Both Dnmt3a and Tet2 have been implemented in controlling adult NSC activity. Dnmt3a can positively control adult neurogenesis. Tet2 has been reported to preserve adult hippocampal NSCs. However, detailed underlying mechanism is still unclear. Moreover, whether Tet2 has DNA demethylation independent function also remains to be explored.

In mammalian CNS, the basic-helix-hoop-helix (bHLH) transcription factors, Olig2 plays a central role in guiding oligodendrocytes and motor neuron development and also shows inhibitory effects on astrocytic differentiation. Spontaneous differentiation of in vitro cultured E11 NSCs can give rise to neurons, oligodendrocytes as well as astrocytes, while forced expression of Olig2 leads to a decrease in the number of astrocyte as well as a decrease in the expression of astroglial genes, such as Gfap, S100b, Id2, and Aqp4. To understand the mechanisms underlying Olig2-dependant repression on astrocytic lineage, we mapped genome-wide Olig2 occupancy in E11 NSCs by ChIP-chip. Mapping Olig2 binding sites to the regions flanking TSS revealed that Olig2 does not physically associated to the promoter of the astrocytic genes, such as Gfap and S100b, despite the fact that it represses expression of astrocyte marker genes. Olig2 binds to Nfia, which are known to play a crucial role in the onset of astroglial development. Taken together, Olig2 inhibits astrocyte differentiation likely via both direct inhibition of Nfia as well as indirectly, via inhibition of Tet2 expression. Together, this study not only revealed a novel function of Tet2 in promoting astrogliogenesis, but also a novel mechanism by which Olig2 inhibits astrocyte differentiation.

## Materials and methods

### Subjects

The mice used in the experiment were CD1 and Dnmt3a^flox/flox^ mice, CD1 mice purchased from Shanghai SLAC Laboratory Animal Co. Ltd. Dnmt3a^flox/flox^ mice (032289) purchased from Jackson Laboratory. Mice were housed four per cage, maintained on a 12-h light/dark schedule, and allowed free access to food and water, following protocols approved by the Animal Research Committee of Tongji University School of Medicine, China.

### Cell culture

Primary neural progenitor cells (NPCs) are prepared from 6-week time-pregnant CD1 mice or Dnmt3a^flox/flox^ mice. Telencephalon was dissected from E11 mice was first coarsely dissociated by mechanical force then treated with Papain (Worthington) for 5 min at 37°C with constant shaking. 3 × 10^6^ dissociated cells were then plated onto a poly-ornithine (Sigma, 15 µg/ml in H_2_O) and fibronectin (Sigma, 2 µg/ml in PBS) coated 10-cm dishes in serum-free medium containing DMEM/F12 (Invitrogen), 1% B27 (Invitrogen), and penicillin-streptomycin (50 µg/ml and 50 U/ml, respectively). Cells were fed with basic fibroblast growth factor (bFGF, PeproTech) at a final concentration of 10 ng/ml on a daily base. NPCs were passaged with enzymatic dissociation using StemPro Accutase (Life Technologies) upon reaching confluency, and re-plated on PO/FN coated plate at a density of 1–2 × 10^6^ cells per 10 cm dish. For NPC spontaneous differentiation, cells were cultured in medium containing DMEM/F12 (Invitrogen), 1% B27 (Invitrogen), and penicillin-streptomycin (50 µg/ml and 50 U/ml, respectively) without bFGF.

### Virus’s infection

Tet2 catalytic domain overexpressing (Tet2OE) viruses and Tet2 shRNA knock-down (Tet2KD) viruses were produced according to work published by Yi Zhang’s group^16^; Olig2 overexpressing (Olig2OE) viruses were produced according to our previous work^11^. Packaged virus particles were harvested at 48 h and 72 h post transfection, then concentrated using ultracentrifugation (Beckman SW28 rotor, 15,000 rpm for 180 min). The embryonic NPC or Dnmt3a^flox/flox^ NPC were infected with concentrated Control or Tet2OE, Tet2KD, Olig2OE, Cre-expressing viruses in presence of 4 μg/ml polybrene (Sigma) in 2 consecutive days, then maintained 10 ng/ml basic fibroblast growth factor (bFGF, PeproTech) another 5 days, and analyzed in accordance with experimental requirements.

### Immunocytochemistry

NPCs were dissociated, plated on coverslips pre-coated with poly-ornithine (Sigma, 15 µg/ml in H2O) and fibronectin (Sigma, 2 µg/ml in PBS) and cultured till confluency or certain days into differentiation. Cells were fixed with 4% formalin/PBS solution at room temperature for 10 min and washed with PBS for three times. Fixed cells were permeabilized using 0.4% Trixon-X(Sigma), incubated with primary antibodies at 4 °C overnight, followed by secondary antibodies at room temperature for 60 min. Cells were washed with PBS for three times after both primary and secondary antibodies. Cells were stained with neuronal marker Tuj1 (Covance; 1:1000), oligodendtocyte marker CNPase (Millipore; 1:1000) or astrocyte marker Gfap (Sigma; 1:1000). Hoechst staining was used to label the nuclei. Images were captured using Olympus fluorescence microscope and processed using Imaris and Adobe Photoshop CS6 software.

### Quantitative RT-PCR

Total RNA was extracted using Trizol (TRIzol, Invitrogen) following manufacturer’s instructions.

Genomic DNA contamination was removed using Turbo DNase (Ambion), and cDNA was synthesized using SuperScript® III First-Strand Synthesis System (Invitrogen) following the manufacturer’s instruction. Quantitative RT-PCR was performed on a StepOnePlus Real-Time PCR System (Life Technologies) using FastSYBR Green Master Mix (Applied Biosystems). Melting curves were analyzed to confirm a single species of each PCR product. Gapdh cDNA was used as an internal control to quantify the relative expression of each cDNA (2^−ΔΔCT^ method). Experiments were repeated in triplicate.

### Western blotting

Tissue or cultured cells were homogenized in lysis buffer containing 10 mM Tris-HCl, pH 8.0, 150 mM NaCl, 1 mM EDTA, 1% Nonidet P-40, 10% glycerol, and protease inhibitor cocktail (Roche). The protein concentration was measured using Pierce BCA Protein Assay Kit (Thermo Scientific) following the manufacturer’s instruction. The lysates (10–25 µg protein per lane) were separated by SDS-PAGE gel (6–10%) and transferred to nitrocellulose membrane (Bio-Rad) for immunoblotting. The primary antibodies used were Tet2 (Santa Cruz, 1:200), Gfap (Sigma; 1:1000) and internal control β-actin (Sigma, 1:3000).Followed by incubation with horseradish peroxidase (HRP)-conjugated secondary antibodies (Santa Cruz Biotechnology). Signal was detected by an ECL kit (Thermo Scientific). The experiments were performed three times.

### Chromatin immunoprecipitation

ChIP-on-chip were carried out using Agilent Mammalian ChIP-on-chip protocol. In brief, 5 × 10^7^ to 1 × 10^8^ mouse NPCs were dissociated and suspended in 10 ml PBS at room temperature. Cells were then chemically crosslinked by adding 1 ml 11% formaldehyde (Sigma) solution containing 50 mM Hepes-KOH, pH7.5, 100 mM NaCl, 1 mM EDTA, pH 8.0 and 0.5 mM EGTA, pH 8.0 to every 10 ml of cell suspension and rotating at room temperature for 10 min. 0.5 ml of 2.5 M glycine (Sigma) was added to neutralize the formaldehyde. Cells were collected by centrifugation at 1350 × *g* for 5 min at 4 °C and washed with cold PBS twice. Pellets were either flash frozen using liquid nitrogen and stored at −80 °C or directly proceeded to the next step. Crosslinked pellets were then lysed using subsequent treatment of lysis buffer 1, 2, and 3 supplemented with protease inhibitor cocktail (Roche). Nuclei pellets were collected by centrifugation and suspended in 0.3 ml lysis buffer 3 with 1% Triton-X (Sigma) and sonicated using Bioruptor (Diagenode). Samples are sheared for 15 rounds of sonication cycles (30 s ON/30 s OFF) at high power setting with the Bioruptor combined with the Bioruptor water cooler (Diagenode), resulting fragments of 400–600 base pairs (bp) in length. Hundred micrograms of sheered chromatin were mixed with 30 µl of Dynabeads (Dynabeads® M-280 Sheep Anti-Mouse IgG or Dynabeads M-280 Sheep anti-Rabbit IgG, Invitrogen) pre-incubated with 2–10 µg of antibodies (mouse/rabbit IgG, Tet2, Olig2) overnight at 4 °C on a rotating platform. The beads were washed three times with RIPA buffer the next day. Chromatin was eluted from beads by incubation in elution buffer containing 50 mM Tris-HCl, pH8.0, 10 mM EDTA, pH 8.0 and 1% SDS at 65 °C for 15 min with brief mixing on vortex. Eluted chromatin and WCE (whole cell extract) were then reverse-crosslinked at 65 °C for 6 h to overnight. IP and WCE were treated with RNase and protease K and purified using phenol-chloroform extraction. Purified DNA was used in chip, sequencing and qPCR in order to identify Tet2 and Olig2 binding sites. The experiments were performed two times.

### MeDIP and hMeDIP

MeDIP and hMeDIP were performed as previously described^35^. For methylated and hydroxymethylated DNA immunoprecipitation (IP), genomic DNA was extracted using phenol-chloroform. One microgram of genomic DNA was used per IP with similar procedure as ChIP described previouslys. Purified genomic DNA was fragmented by Covaris (Covaris) and mixed with 1 µg of 5mC (eurogentec) and 5hmC (Active motif) antibody conjugated with Dynabeads®M-280 Sheep Anti-Rabbit IgG (Invitrogen) respectively. DNA fragments pulled down from MeDIP and hMeDIP, as well as genomic DNA (input) were end-repaired by T4 DNA polymerase and phosphorylated. A single ‘A’ base was added to the 3′ end with Klenow. Adaptors with indexes were ligated to the fragments with multiplexing sample preparation kit (Illumina). Ligation products between 300 and 500 bp were purified using AMPure beads (NEB) and amplified by PCR. Libraries were quantified with PicoGreen and QC with Bioanalyzer then analysed by Illumina Hiseq2000 platform. The experiments were performed three times.

### ChIP targets validation

Site-specific primers were designed for Olig2 and Tet2 binding sites and methylation/hydroxymethylation sites identified from ChIP-chip, ChIP-seq or adopted from previously published studies. Quantitative PCR was performed on a StepOnePlus Real-Time PCR System (Life Technologies) using Fast SYBR Green Master Mix(Applied Biosystems). Fold enrichment were calculated by IP over IgG or IP over WCE. The experiments were performed three times.

### Whole-genome expression analysis

mRNA library was prepared using NEBNext Utra mRNA Library Prep Kit for Illumina (NEB) following manufacturer’s protocol. Briefly, total RNA was extracted following manufacturer’s instructions (TRIzol, Invitrogen). mRNA was isolated using NEBNext Poly (A) mRNA Magnetic Isolation Module (NEB) and fragmented using Covaris (Covaris). cDNA was synthesized using random priming, followed by end repair and 5′ phosphorylation. DA-tailing was added and adaptors with indexes were ligated. Ligation product was amplified using PCR, products between 300 and 500 bp were purified using AMPure beads (NEB) and amplified by PCR. Libraries were quantified with PicoGreen and QC with Bioanalyzer before analyzed by Illumina Hiseq2000 platform.

### Data analysis

#### Data QC, trimming, and mapping

FASTQ files were generated from HiSeq2000 with at ~25 million reads (50 bp) per lane. Reads were first de-multiplexed according to the corresponding indexes. Quality control was performed using FastQC (The Picard BAM/SAM Libraries), the indexes and base sequences with a quality score below 20 are removed.Trimmed reads were aligned to mouse reference genome (MM10) using TopHat and Bowtie2^36–38^ with default settings (allow maximum two mismatches).

### RNA-seq analysis

Reads were mapped to the mouse genome (mm10) using Bowtie and TopHat using default parameters. BAM files generated from mapping were then submitted to Cuffdiff for differential gene expression detection. Results were visualized using UCSC Genome Browser and CummeRbund^39^.

### ChIP and MeDIP/hMeDIP data analysis

Reads were mapped to the mouse genome (mm10) using Botwie2. Binding peaks were identified using MACS^40^ with default settings and visualized using UCSC Genome Browser^39^. MeDIP and hMeDIP data were first analyzed using MEDIPS with the parameters suggested in the manual^41^. The average signal enrichment of methylation/hydroxymethylation at TSS was plotted using SitePro from the CEAS (Cis-regulatory Element Annotation System) with a profiling resolution of 100nt and spanning 1500 bp of the TSS region^42,43^.
